# Supplementary material for: Ship wake-induced water column mixing and meter-scale seabed erosion in the Baltic Sea
Source: Nat Commun. 2026 Feb 10;17:1350. doi: 10.1038/s41467-026-68875-6 (PMC12891571; doi:10.1038/s41467-026-68875-6)
Supplement: Supplementary file 1 — Reporting Summary [file 41467_2026_68875_MOESM1_ESM.pdf]

nature portfolio

Corresponding author(s): Jacob Geersen

Last updated by author(s): Nov 25, 2025

Reporting Summary

Nature Portfolio wishes to improve the reproducibility of the work that we publish. This form provides structure for consistency and transparency in reporting. For further information on Nature Portfolio policies, see our Editorial Policies and the Editorial Policy Checklist.

Statistics

For all statistical analyses, confirm that the following items are present in the figure legend, table legend, main text, or Methods section.

n/a	Confirmed	
☐	☒	The exact sample size (*n*) for each experimental group/condition, given as a discrete number and unit of measurement
☒	☐	A statement on whether measurements were taken from distinct samples or whether the same sample was measured repeatedly
☒	☐	The statistical test(s) used AND whether they are one- or two-sided<br>*Only common tests should be described solely by name; describe more complex techniques in the Methods section.*
☒	☐	A description of all covariates tested
☒	☐	A description of any assumptions or corrections, such as tests of normality and adjustment for multiple comparisons
☒	☐	A full description of the statistical parameters including central tendency (e.g. means) or other basic estimates (e.g. regression coefficient) AND variation (e.g. standard deviation) or associated estimates of uncertainty (e.g. confidence intervals)
☒	☐	For null hypothesis testing, the test statistic (e.g. *F*, *t*, *r*) with confidence intervals, effect sizes, degrees of freedom and *P* value noted<br>*Give P values as exact values whenever suitable.*
☒	☐	For Bayesian analysis, information on the choice of priors and Markov chain Monte Carlo settings
☒	☐	For hierarchical and complex designs, identification of the appropriate level for tests and full reporting of outcomes
☒	☐	Estimates of effect sizes (e.g. Cohen's *d*, Pearson's *r*), indicating how they were calculated

Our web collection on statistics for biologists contains articles on many of the points above.

Software and code

Policy information about availability of computer code

Data collection	No software was used for collection of data.
Data analysis	ArcGIS for morphological analysis<br>QPS for processing of the multibeam and split-beam data

For manuscripts utilizing custom algorithms or software that are central to the research but not yet described in published literature, software must be made available to editors and reviewers. We strongly encourage code deposition in a community repository (e.g. GitHub). See the Nature Portfolio guidelines for submitting code & software for further information.

Data

Policy information about availability of data

All manuscripts must include a data availability statement. This statement should provide the following information, where applicable:

- Accession codes, unique identifiers, or web links for publicly available datasets
- A description of any restrictions on data availability
- For clinical datasets or third party data, please ensure that the statement adheres to our policy

The bathymetry data of 2014 were collected by BSH and are freely available via the SeaDataNet Pan-European infrastructure for ocean and marine data management (https://www.seadatanet.org). Automatic identification system (AIS) data are archived by the Danish Maritime Authority (https://www.dma.dk), which provides free access to historical AIS raw data and information on AIS usage. The 2024 bathymetric and EK60 data are stored by the Marine Geophysics and Hydroacoustics working group at Kiel University and will be made available from the authors on reasonable request, see author contributions for specific data

sets.

Research involving human participants, their data, or biological material

Policy information about studies with human participants or human data. See also policy information about sex, gender (identity/presentation), and sexual orientation and race, ethnicity and racism.

Reporting on sex and gender	We only use geophysical and sedimentological data.
Reporting on race, ethnicity, or other socially relevant groupings	We only use geophysical and sedimentological data.
Population characteristics	We only use geophysical and sedimentological data.
Recruitment	We only use geophysical and sedimentological data.
Ethics oversight	We only use geophysical and sedimentological data.

Note that full information on the approval of the study protocol must also be provided in the manuscript.

Field-specific reporting

Please select the one below that is the best fit for your research. If you are not sure, read the appropriate sections before making your selection.

☐ Life sciences ☐ Behavioural & social sciences ☒ Ecological, evolutionary & environmental sciences

For a reference copy of the document with all sections, see nature.com/documents/nr-reporting-summary-flat.pdf

Ecological, evolutionary & environmental sciences study design

All studies must disclose on these points even when the disclosure is negative.

Study description	We measured the structure and morphology of the seafloor and the overlying water column.
Research sample	We use marine geophysical data. The data is stored on hard drives.
Sampling strategy	We measured the structure and morphology of the seafloor in an area of about 30 km^2. The size of the measured area was chosen to being able to resolve regional seafloor morphology.
Data collection	Multibeam bathymetric data analysed in this manuscript were collected in 2014 by the German Federal Maritime and Hydrographic Agency (BSH), and 2024 by the Marine Geophysics and Hydroacoustic Group of Kiel University during RV ALKOR cruise AL619.
Timing and spatial scale	see above
Data exclusions	No data were excluded
Reproducibility	The datasets cover the same area and we interpret the morphologic changes that occured over 10 years.
Randomization	The data were grouped into two groups depending on the year in which they have been collected (2014 and 2024).
Blinding	Not relevant because we solely use geophysical and sedimentological data.

Did the study involve field work? ☒ Yes ☐ No

Field work, collection and transport

Field conditions	good weather and medium wave heights
Location	Baltic Sea, Kiel Bay, German waters
Access & import/export	No permits were required for the collection of the data. However, we followed the convention for responsible marine research as defined by the DFG Senatskommission für Ozeanographie and the KDM, which were accepted without reservations and fully implemented.
Disturbance	No disturbance

Reporting for specific materials, systems and methods

We require information from authors about some types of materials, experimental systems and methods used in many studies. Here, indicate whether each material, system or method listed is relevant to your study. If you are not sure if a list item applies to your research, read the appropriate section before selecting a response.

Materials & experimental systems

n/a	Involved in the study
☒	☐ Antibodies
☒	☐ Eukaryotic cell lines
☒	☐ Palaeontology and archaeology
☒	☐ Animals and other organisms
☒	☐ Clinical data
☒	☐ Dual use research of concern
☒	☐ Plants

Methods

n/a	Involved in the study
☒	☐ ChIP-seq
☒	☐ Flow cytometry
☒	☐ MRI-based neuroimaging

Plants

Seed stocks	No seed stocks used
Novel plant genotypes	No novel plant genotypes used
Authentication	No authentication necessary
